# 3D Printed Multimaterial Microfluidic Valve

**DOI:** 10.1371/journal.pone.0160624

**Published:** 2016-08-15

**Authors:** Steven J. Keating, Maria Isabella Gariboldi, William G. Patrick, Sunanda Sharma, David S. Kong, Neri Oxman

**Affiliations:** 1 Media Lab, Massachusetts Institute of Technology, Cambridge, Massachusetts, United States of America; 2 Lincoln Laboratory, Massachusetts Institute of Technology, Lexington, Massachusetts, United States of America; Universita del Salento, ITALY

## Abstract

We present a novel 3D printed multimaterial microfluidic proportional valve. The microfluidic valve is a fundamental primitive that enables the development of programmable, automated devices for controlling fluids in a precise manner. We discuss valve characterization results, as well as exploratory design variations in channel width, membrane thickness, and membrane stiffness. Compared to previous single material 3D printed valves that are stiff, these printed valves constrain fluidic deformation spatially, through combinations of stiff and flexible materials, to enable intricate geometries in an actuated, functionally graded device. Research presented marks a shift towards 3D printing multi-property programmable fluidic devices in a single step, in which integrated multimaterial valves can be used to control complex fluidic reactions for a variety of applications, including DNA assembly and analysis, continuous sampling and sensing, and soft robotics.

## Introduction

The introduction of the first valves fabricated via soft lithography [1] ignited the field of microfluidics, enabling device programmability and automation at a previously unprecedented scale [2]. The simplicity and ease of soft lithography increased the accessibility of programmable microfluidics to non-experts in the area of microfabrication, engaging researchers from diverse fields of science and engineering [3]. However, notable limitations to soft lithography remain. The two-dimensional nature of replica molding makes the production of 3D structures beyond several layers extremely challenging, curtailing the capabilities and therefore the applications associated with the technique. Furthermore, photolithography and the production of molds for replica molding require infrastructure for realization of physical products.

In recent years, advances in Additive Manufacturing (AM) have enabled new and interesting opportunities associated with the design and digital fabrication of geometrically complex and materially heterogeneous objects with high spatial resolution. For example, AM tools have been implemented to create microfluidic devices. 3D printed milli- and microfluidic systems have been fabricated through fused-deposition modeling (FDM) of thermoplastics [4], as well as stereolithography and inkjet printing of photo-curable polymers [5–7]. Fabrication of digitally designed parts can occur within hours, compared to days for soft lithography [8]. 3D design files can easily be shared online, allowing for collaboration on new designs that can be 3D printed on site or mail-ordered at low costs [8,9].

3D printing also offers a variety of technical advantages over replica molding. Conventional soft lithography techniques limit achievable microfluidic geometries to two-dimensional (planar) molded forms. Tedious multi-step processes, such as layer-by-layer fabrication, are required to produce 3D geometries. More recently, 3D printing has been used to produce scaffolds with complex geometries that can be embedded in polydimethylsiloxane (PDMS) and subsequently dissolved, leaving behind channels in the shape of the original 3D printed structure [10]. While resolution is still limited, 3D printing enables the design and construction of complex geometries through single-step fabrication [11]. 3D printed materials can also be tailored for specific applications [12–16], circumventing some of the traditional material limitations posed by PDMS, such as incompatibility with many solvents [17].

Currently, most 3D printed microfluidics are designed and fabricated as passive devices with limited fluid handling capability and programmability [18]. Most recently, 3D printed valves for microfluidic devices have been created that are made of a single material and are actuated via membrane deformation through applied fluidic pressure [19–21]. However, homogeneous material valves are large in size (typical radius of 5 mm [19]) and exhibit global deformation during actuation that restricts device complexity and size.

Multimaterial 3D printing of valves can be used to fabricate devices that not only overcome these drawbacks but also offer new functionalities. Current multimaterial 3D printers use materials with variable properties such as stiffness, opacity, and color [22]. Single parts can therefore be printed out of multiple materials, with pre-set mechanical and optical properties, and their combinations. This fabrication method has previously been used to print parts with stiffness gradients ranging from rubber-like to hard plastic [23]. We hypothesized that a similar method could be used to create valves by printing hard channel sections separated by flexible layers.

## Materials and Methods

**Fig 1:**
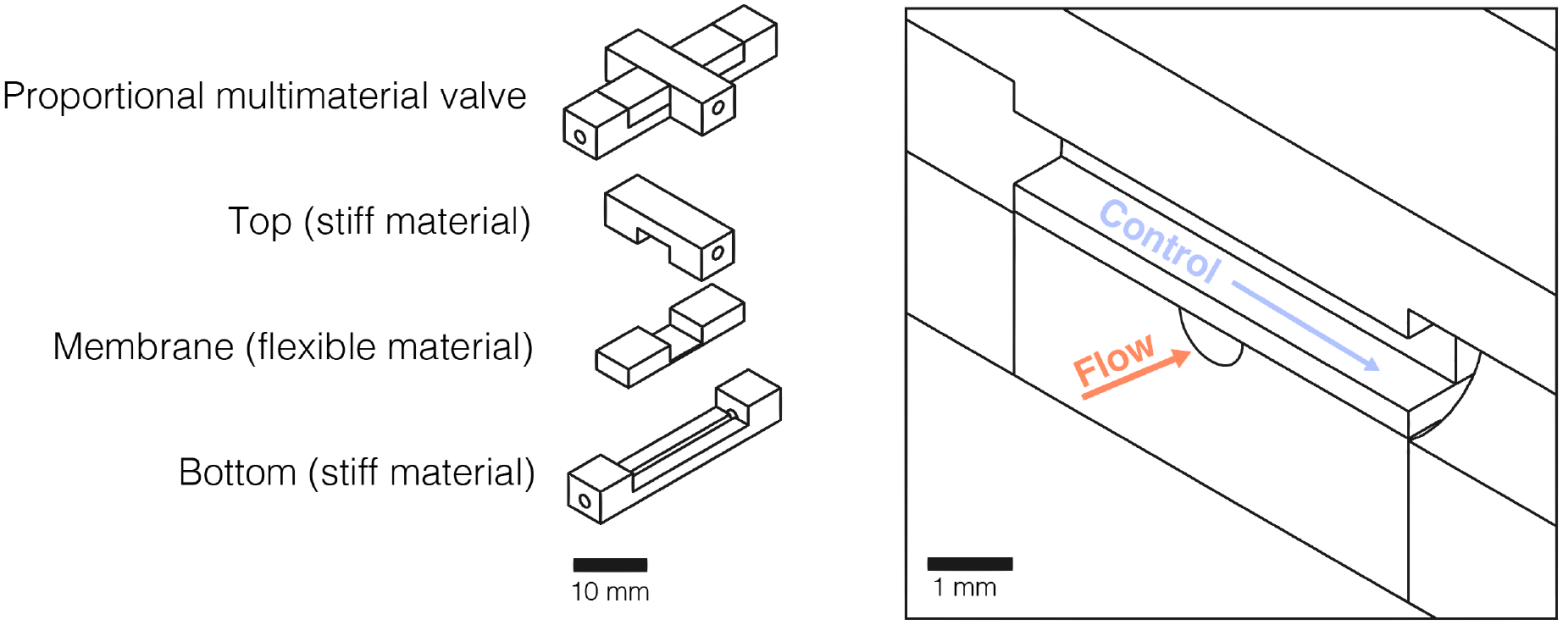
Multimaterial valve design. Design and material breakdown (left). Assembled valve cross-section showing the control and flow channels (right). Applied fluidic pressure to the control channel actuates the valve by deforming the flexible membrane to obstruct the flow channel.

Microfluidic valves were designed using the computer-aided design (CAD) software, SolidWorks (Dassault Systèmes Americas, Waltham, MA), with a geometry modeled after traditional push-up microfluidic valves [1, 24]. Single material push-up valves have been widely used and the geometry was chosen to develop a proof-of-concept 3D printed multimaterial valve. Push-up valves comprise a semi-circular ‘flow channel’ and a rectangular ‘control channel’ separated by a membrane (Fig 1). They operate like those developed by Unger et al. [1]: when pressure is applied to a control channel, the membrane deflects, sealing the flow channel. The valves were 3D printed with a flexible material for the membrane and a stiff material for the external valve structure, in order to reduce global deformation and facilitate valve tuning for different pressures. Dimensions and materials were varied per Table 1, with a standard membrane thickness of 300 μm, control channel width of 800 μm, control channel height of 800 μm, and a semi-circular flow channel radius of 400 μm. Internal tube ports to all channels were printed with a radius of 750 μm in order to fit tubing with an external diameter of 1500 μm. For more information on the physical valve design, please see the Supporting Information for computer design files (in STL file format) of the standard dimension 3D printed multimaterial valve used in this research.

**Table 1:** Requisite Closure Pressures.

	Parameter Design Variations (all other parameters are held constant according to the standard design)										
	Membrane Thickness				Channel Width				Membrane Material Stiffness		
	200 μm	300 μm ^a^	400 μm	500 μm	700 μm	900 μm	1100 μm	1300 μm	Shore 26–28 Scale A^a^	Shore 35–40 Scale A	Shore 57–63 Scale A
Control Pressure for Valve Closure (defined as ≤ 3.5 μL/s full flow) (psi)	15.0	28.8	36.3	42.5	30.0	26.3	25.0	22.5	28.8	35.0	>73.0^b^

Control pressure required for valve closure experimentally found for design variations. Listed stiffness values for the membrane material are sourced from Stratasys, Ltd material data sheets [24]. Closing pressures varied between different samples or different runs. The value represents the average control pressure at which the valve was closed (defined by a flow rate ≤ 3.5 uL/s) for all trials and printed valves. Error of ± 4.4 psi was found between trials of the same printed valve.

^a^ Same sample data

^b^ Valve did not close at control pressures tested. Flow measured at 73.4 psi was ~49% total flow and a single trial was conducted for this file.

All devices were printed using a Stratasys Objet500 Connex multimaterial 3D printer [24]. This printer operates using the Stratasys PolyJet printing technology: droplets of photo-curable liquid polymer are jetted and cured with ultraviolet light, producing ‘digital materials’ made spontaneously by mixing different liquid polymers in known pre-set mechanical combinations. Based on the materials commercially available for the printer, the valves were fabricated with the most flexible material (TangoPlus, FLX930) and a stiff material (VeroWhitePlus, RGD835) [25]. TangoPlus is a rubber-like translucent material and VeroWhitePlus is a rigid opaque white material. The fabrication workflow utilized these two materials to create digitally mixed materials with different tensile strength/tear resistance and translucency based on different proportions of the cartridge materials deposited by the printer. For our printed valves, the printer deposits 30 μm thick layers with gel-like support material to reinforce hollow structures and overhangs while printing. Support material was manually cleared from all internal features using pressurized air and a metal rod. External tubing was inserted in the printed valve ports and sealed with Sil-Poxy silicone adhesive.

**Fig 2:**
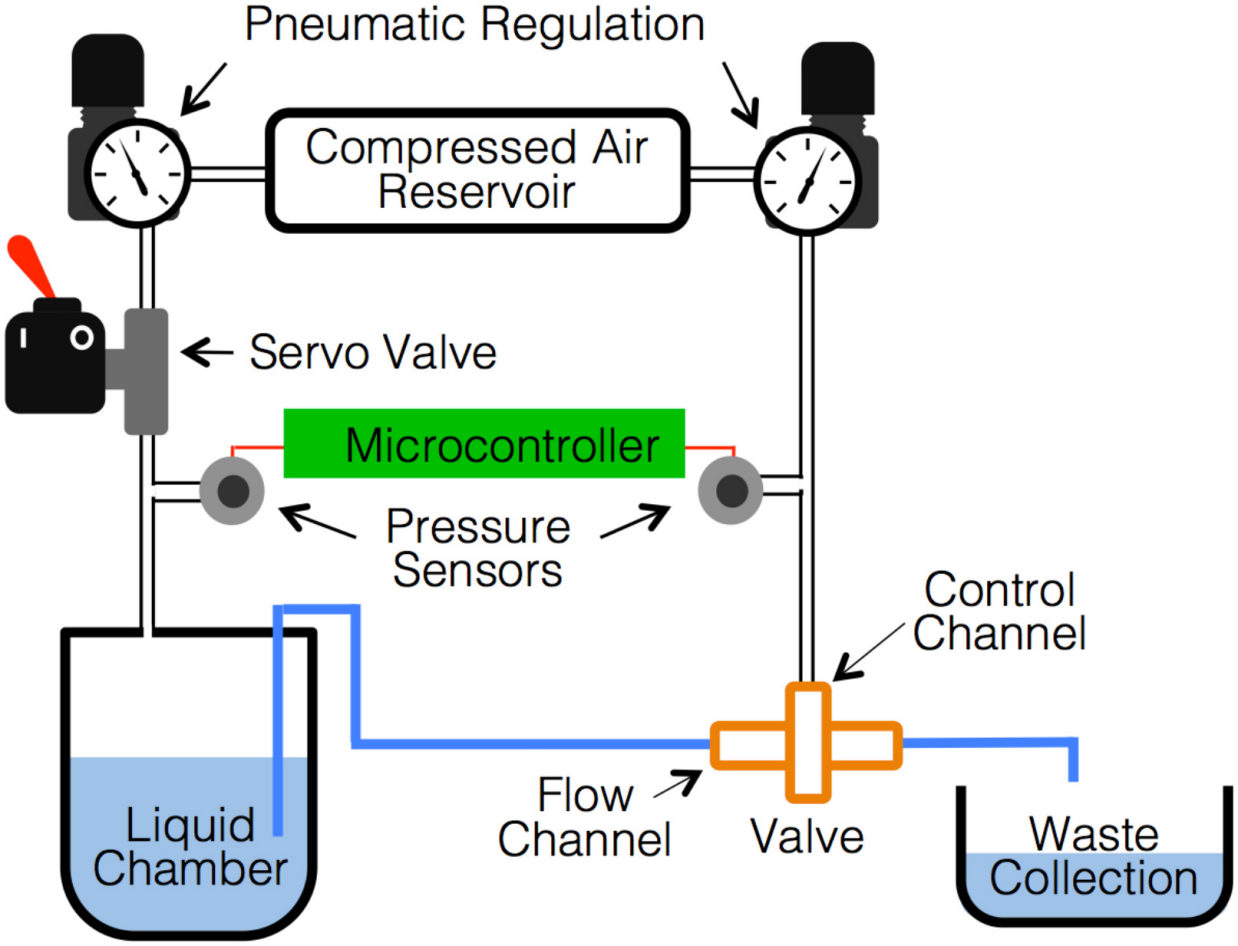
Experimental setup used to characterize printed fluidic valves. Pneumatic regulators were used for control channel actuation and to pressurize the liquid chamber along with solenoid valves. Pressure sensors connected to an Arduino UNO microcontroller were used to record both control channel and liquid chamber pressures.

Fabricated valves were characterized using a measurement system built in-house, as shown in Fig 2. A pressure sensor (MPX5700AP, Freescale Semiconductor, Austin, TX, 2.5% maximum error) was used to measure applied pressure in the control channel alongside a visual pressure gauge. A barometric pressure sensor (BMP180, Bosch Sensortec, Reutlingen, Germany, maximum error of 0.06 psi) was placed in line with a liquid chamber containing a fixed initial volume of water. Tubing was used to connect the liquid chamber to the flow channel, such that upon pressurization of the vessel, the system was completely sealed when the valve was closed. The liquid chamber was pressurized at around 2 psi to initiate fluid flow and the rate-of-change in pressure measured by the barometric pressure sensor was used to calculate fluid flow through the valve using Boyle’s Law. Both pressure sensors were connected to an Arduino UNO microcontroller for recording data and the system temperature was maintained at room temperature (22°C) throughout the experiment. The liquid chamber and control channel pressures were controlled using pneumatic elements and solenoid valves. Prior to collecting data for each valve, the control line was pressurized to completely close the valve for 2 minutes to ensure full membrane deformation after printing. Data was collected in trial runs, incrementally increasing control line pressures, starting at atmospheric pressure. Between each increment in control pressure, the liquid chamber pressure was brought back to 2 psi before beginning each measurement trial. For more information on the experimental workflow, trial data, and data calculations, please see the Supporting Information.

**Fig 3:**
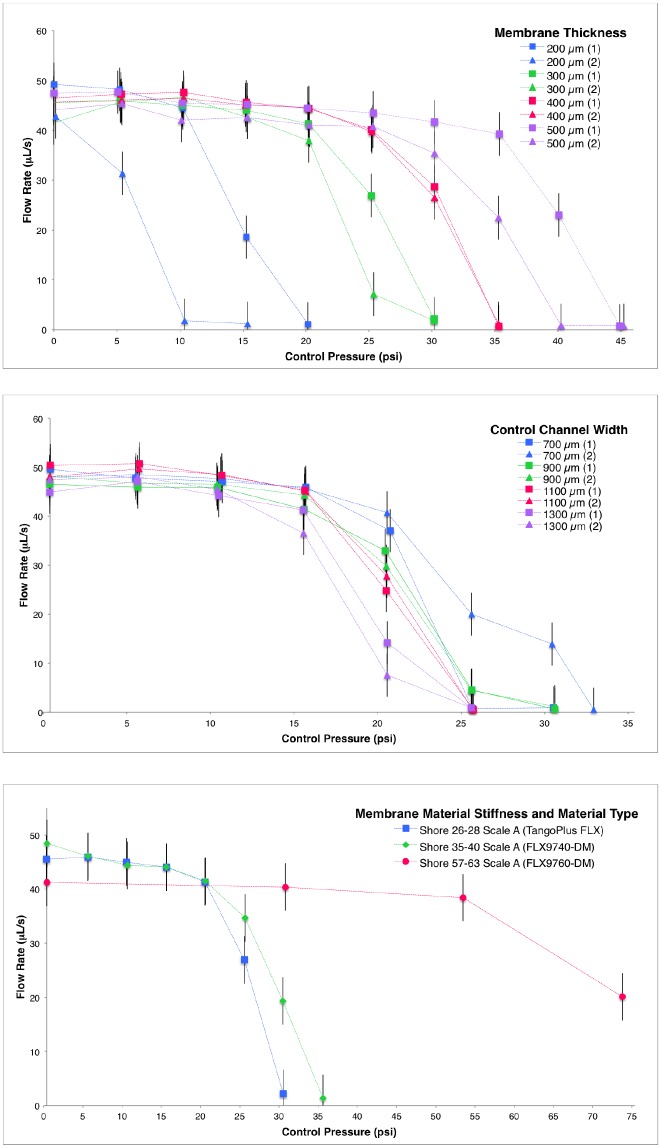
Fluid flow under various conditions. Measurements of fluid flow through the 3D printed multimaterial valve with varying applied pressure to the control channel are plotted. Dimensions and materials were varied according to Table 1, with a standard membrane thickness of 300 μm, control channel width of 800 μm, control channel height of 800 μm, and a semi-circular flow channel radius of 400 μm. Proportional control of the flow rate is demonstrated and the design parameters are explored through varying membrane thickness (top), control channel width (middle), and membrane material (bottom). Connecting lines are shown only for visualization and the charted points and error bars represent the measured data. For membrane thickness and control channel width, two prints of each design file were tested and each is plotted using the average of two trials per print. The bracketed number in the legend indicates a different print of the same design file and both are plotted for clarity with the same color. Membrane material type tests used a single print. Error bars show repeatability between trials at the same pressure for different prints of the same valve specifications (± 4.4 psi, calculated with 95% confidence interval). Listed stiffness values for the membrane material are sourced from Stratasys, Ltd. Material data sheets [25]. Please see the Supporting Information for all of the data from the trials.

The system measurement error was experimentally found using a precision scale (Mettler Toledo) to compare the weight of the total liquid flow against the calculated flow measured through the change in pneumatic pressure in the liquid chamber during calibration trials (error of ± 3.4%). Two measurement trials at each pressure for the same valve were conducted to measure functional repeatability of a single printed piece (error of ± 4.4 psi, based on 95% confidence interval, from comparing a total of 222 flow measurements). The average flow measurement value from the two trials for each control pressure tested was plotted (Fig 3, Table 1) with ± 4.4 psi error bars. Multiple valves were printed from the same design file to quantify differences in functionality due to the manual support cleaning process and overall repeatability of the process.

## Results and Discussion

3D printed multimaterial valves were successfully fabricated, demonstrating proportional control of the flow channel when air pressure was applied to the control channel. Each design was printed with variations in channel width, membrane thickness, and membrane stiffness, as seen in Table 1. Valves were first tested to determine the required closing pressure for each design. As expected, the required pressure to close the valve was higher in designs with relatively narrower control channels, larger membrane thicknesses, and stiffer materials (Table 1). Only three of nine material types tested as membrane materials were flexible enough to deform and function properly with the standard design geometry used. The print time for a single valve was 25 minutes. Multiple valves can be printed in parallel on the same print tray to reduce fabrication time for larger batches. Each valve used 2 grams of stiff material, 2 grams of flexible membrane material, and 4 grams of support material, totaling a per part material cost of $1.85 USD [26]. While the multimaterial 3D printer used for valve fabrication is an expensive and high performance machine, the low material cost per microfluidic device allows for future accessibility through distributed fabrication spaces, such as a TechShop location [27], or consumer-friendly online 3D fabrication services like Shapeways [28] or i.materialise [29], where custom parts can be printed for a fee without ownership of a printer.

Characterization results for the printed valve are graphically shown in Fig 3. The results show proportional control over the valve flow rate ranging between 0 and 50 μL/s, as well as trends in the data due to dimensional variations and variations in material properties. Increased membrane thickness–between 200 μm and 500 μm–resulted in higher required control pressures, ranging between 15 psi and 43 psi for a full closure. With wider control channels–between 700 μm and 1300 μm–lower control pressures were required, ranging between 22.5 psi and 30 psi. Finally, for increasing membrane material stiffness–between Shore 26–63 Scale A–higher control pressures were required (between 29 psi and 73 psi).

**Fig 4:**
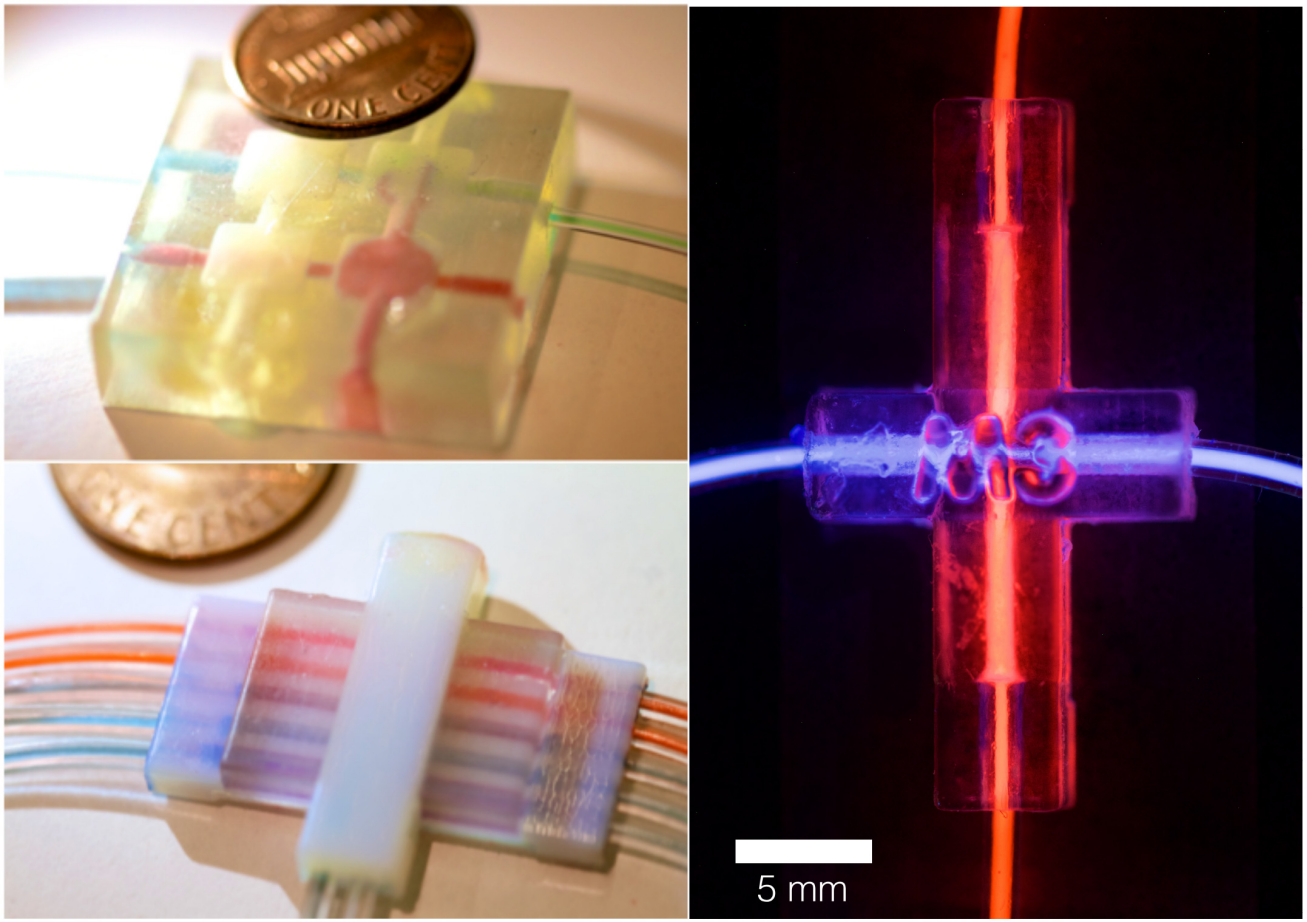
3D printed microfluidic valves. A) A 3D printed single material multichannel valve system (top left) and a 3D printed multimaterial multichannel valve system (bottom left). The single material valve contains two control channels with square chambers both above and below the flow channels and the multimaterial valve uses the same design methodology previous described in the paper. For the multimaterial multichannel valve, all channel valves are actuated at the same time by the control line. The global deformation of the single material valve system is significantly larger than the multimaterial valve during actuation, as seen in S1 Video B) A 3D printed multimaterial valve with chemiluminescent liquid in the control and flow channels for visualization (right).

Eliminating material deformation during actuation beyond the valve design geometry is of critical importance when constructing systems featuring a high density of valves. In operating the multimaterial valve, there was no visual global deformation due to the applied control pressure during actuation. The membrane deformation during actuation is a local deformation that is constrained by the stiff surrounding material. In contrast, we printed a multichannel single material valve (Fig 4) that demonstrated large global deformations easily visible by eye (S1 Video).

During fabrication and testing, we discovered an important factor that can lead to variability in performance between printed valves based on the same design file: inconsistent clearing of support material. Support material, in the form of a gel, was manually removed. Significant care had to be taken during cleaning to avoid small tears in the membrane. Water jet cutting and soaking in sodium hydroxide were tested as alternative support material removal techniques. However, both methods were found to significantly increase the likelihood of valve membrane tearing. Any support material that was not removed could impede fluid flow and cause variation in valve performance. Additionally, because an external tool is required to remove this type of support material, there is a limitation to the curvature, complexity, and dimensions of internal channels printed with the technique; straight channels are easier to clear fully due to the tool shape. Repeated use of the device directly after cleaning reduces variability and leads to more consistent performance, but it is conceivable that after a certain point, accumulated wear on the valve will compromise performance.

Secondly, we found that the initial pressurization of the valve resulted in a small variation in flow rate compared to future pressurization trials. We believe the first pressurization removes some additional support material that causes this variation. To reduce this error, before all data trials, we initially pressurized the control line of each valve for two minutes before releasing pressure and beginning testing. Once this initial pressurization was conducted, repeatable proportional control of the same printed valve was shown within the error bounds of ± 4.4 μL/s (95% confidence interval).

## Future Research Directions

**Fig 5:**
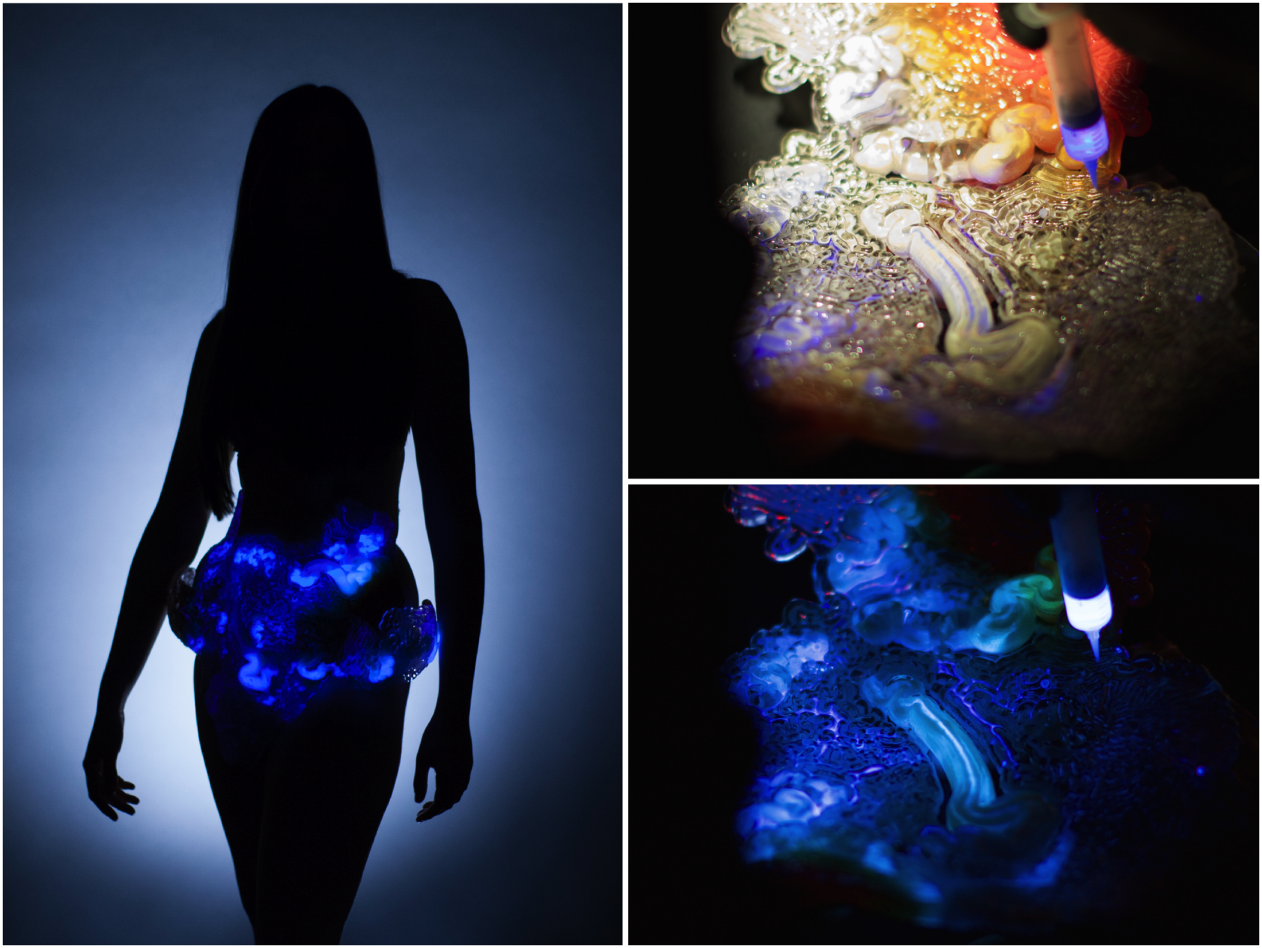
A product-scale wearable millifluidic system 3D printed with an experimental liquid support technique. The digitally designed and fabrication system was printed with colleagues [32] and future work will look to further characterize this experimental printing method. For the images, a chemiluminescent liquid was pumped through the flow channels for visualization.

In this paper, we demonstrated the digital fabrication and characterization of 3D printed multimaterial proportional valves, with flexible membranes constrained by stiffer structural flow and control channels. Printed multimaterial valves can exploit variable material properties for enhanced functionality. For example, we have shown how printed multimaterial valves can reduce global deformation during valve actuation through variable material stiffness. By reducing actuation deformation, multiple independently controlled valves can be spatially located in close proximity in a printed system, allowing for complex programmable microfluidic devices. In order to scale the process and 3D print additional complex fluidic devices in large quantities, we must overcome the primary limitation encountered in this work: manual removal of support material [30]. Two alternative support methods, namely, dissolvable wax support and liquid support, hold the potential of overcoming this limitation [31]. Initial investigations of both dissolvable and liquid support materials are being implemented in concurrent work alongside colleagues [32] to create millifluidic channels, also using the Objet500 Connex. Early results point towards exciting potentials, such as a successfully digitally designed and fabricated product-scale wearable fluidic system. Through an experimental liquid support technique, the printed fluidic system in Fig 5 is comprised of a 58-meter long internal channel with variations in diameter, membrane thickness and material properties [32]. In the context of future work, additional characterization and development of such new support methods could enable 3D printing of fluidic devices with integrated valves and detailed internal geometries for novel functionality as well as product applications. For example, multimaterial valves in future wearable devices could control complex fluidic mixing, sensing, filtering, and computation. Additional valve testing is required for long-term use, including; measurements of the durability of the flexible membranes following repeated deformations; functional valve cycle lifetime; compatibility of the materials with commonly used microorganisms and biochemistries; and fidelity of prints with different rigidities and patterning to include biocompatible, semi-permeable, or other materials.

Accordingly, the software component of design will require improvements, such as the inclusion of finite element analysis for optimizing geometries for fluidic devices. Further developments can be made in the manufacturing process to analyze the robustness over printing cycles, the precision depending on liquids used, relation between liquid viscosity and exerted control, and characterizing the range of different material properties and printers that can be used for similar microfluidics protocols.

For applications requiring microscopy, it is desirable for programmable 3D printed fluidic devices to be interfaced with glass microscope slides. In an initial study, we exposed 2 cm slabs of digitally mixed materials from Table 1 with oxygen plasma (Gasonics) at 300W for 30s, a common treatment for generating covalent bonding between glass and PDMS. Each slab also featured a fluidic channel, 1.6 cm long and 1 mm wide. While applying oxygen plasma treatment did not produce covalent bonds to glass, it was still possible to adhere digitally mixed materials to glass substrates. Slabs composed of mixtures containing higher concentrations of TangoPlus, when clean and free of particulates on the surface, could adhere to glass and maintain fluid pressures in the range of 5–10 psi—a common operating pressure in microfluidics—without generating leaks. Further exploration and characterization of surface treatments and pressure-based bonding techniques are warranted to determine methods for generating bonding between 3D printed structures and glass capable of sustaining fluid pressures greater than 10 psi.

Results shown carry significant potential for future developments of 3D printed microfluidics with enhanced custom functionality. Compared with conventional fabrication techniques, this manufacturing process lends itself to rapid scalability through automation, paving the way to new applications in a variety of fields. For example, pumping valves could be used in muscle contraction simulations, emulating peristalsis; cell culture devices could have patient-specific stiffness areas for tissue growth and sorting; controllable soft and bio-inspired robotics could be produced without assembly; integrated and highly controllable sensors could provide continuous sampling; and general pneumatic systems could have new dimensions such as chemical-material gradients and integrated actuation.

## Supporting Information

S1 Additional Files Computer-aided Design Files. Digital design files (STL format) of the standard dimension multimaterial valve are provided to allow replication, usage, and further development of 3D printed valves.
(ZIP)

S1 Appendix Pressure Sensor Data Analysis. Details of how barometric pressure sensor data was analyzed, both to convert rate of fluid chamber pressure change to flow rate, and to generate the plots shown.
(DOCX)

S1 Fig Examples of measurement workflow. Plotted linear fits of parsed data from liquid chamber pressure at full flow (top) and at valve closing at an absolute pressure of 29.66 psi (left) are shown as examples for the measurement workflow. The plotted data is from Sample 1, run 1 of a printed valve with a membrane thickness of 200 μm (it is the first entry in S1 Table below). For the full flow rate as seen in the top plot, the fitted linear trendline has a slope of -2.458E-04 psi per data point, with a R^2^ value of 0.9996. For the bottom plot, the fitted trendline slope is -5.380E-06 psi per data point, with a R^2^ value of 0.5731 (a low R^2^ value as expected due to valve closure resulting in a near-horizontal trendline). Graph pressures plotted as absolute values.
(TIF)

S1 Table Data Processing for Flow Rates for Membrane Thickness. (PDF)

S2 Table Data Processing for Flow Rates for Channel Width. (PDF)

S3 Table Data Processing for Flow Rates for Membrane Material. (PDF)

S1 Video Demonstration video of 3D Printed Single Material and Multimaterial Valve. (MOV)
