# Supplementary material for: 3D Printed Multimaterial Microfluidic Valve
Source: PLoS One. 2016 Aug 15;11(8):e0160624. doi: 10.1371/journal.pone.0160624 (PMC4985141; doi:10.1371/journal.pone.0160624)
Supplement: S1 Appendix — Details of how barometric pressure sensor data was analyzed, both to convert rate of fluid chamber pressure change to flow rate, and to generate the plots shown. (DOCX) [file pone.0160624.s002.docx]

**S1 Appendix**

**Supporting Information**

3D Printed Multimaterial Microfluidic Valve

Steven J. Keating^1¶^, Isabella Gariboldi^1¶^, William G. Patrick^1^, Sunanda Sharma^1^, David S. Kong^2^, and Neri Oxman^1*^

^1^ Media Laboratory, Massachusetts Institute of Technology, Cambridge, Massachusetts, United States of America

^2^ Lincoln Laboratory, Massachusetts Institute of Technology, Lexington, Massachusetts, United States of America

* Corresponding author

E-mail: neri@mit.edu

^¶^These authors contributed equally to this work.

**Conversion of Rate of Fluid Chamber Pressure Change to Flow Rate**

Output data from the barometric pressure sensor was parsed to generate different plots for each applied pressure. The rate of change in the barometric pressure sensor measurements in the fluid chamber was found using a linear fit (Figure A). For each applied pressure, the rate of change in pressure was converted to a flow rate using Boyle’s law. According to Boyle’s Law, the product of the pressure and the volume of an ideal gas is constant in a closed system at a constant temperature and fixed amount of gas. Therefore,

$$V_{t=1}=\frac{P_{t=0}V_{t=0}}{P_{t=1}}$$

where $P_{t=0}$ is the initial air pressure of the liquid chamber (16.7 psi or 115.14 kPa) , $V_{t=0}$ is the initial air volume, $P_{t=1}$ is the air pressure after one second and $V_{t=1}$ is the air volume after one second. $V_{t=0}$ was found to be 0.000173 m^3^ when validating the apparatus using a high precision scale.

The pressure after one second is

$$P_{t=1}=P_{t=0}+\left( m*k \right)t$$

where$t=1$ second, $m$ is the slope of the liquid chamber pressure drop as measured by the barometric pressure sensor and $k$ is the time rate of data measurement of the system (sensor logging data through an Arduino Uno with a serial interface), experimentally measured at 17.11 Hz (data points per second).

The final volume $V_{t=1}- V_{t=0}$ gives the increase in air volume in one second. The change in air volume in the liquid chamber per second is equal to the change in liquid volume due to fluid flowing out of the chamber (flow rate). Tables A, B and C summarize data processing for membrane thickness, channel width and membrane material variations respectively. Generally, linear fits exhibited high R^2^ values except for fits for data collected at pressures close to valve closure. This is because at these pressures, lines were close to horizontal (which, by definition, results in low R^2^ values).


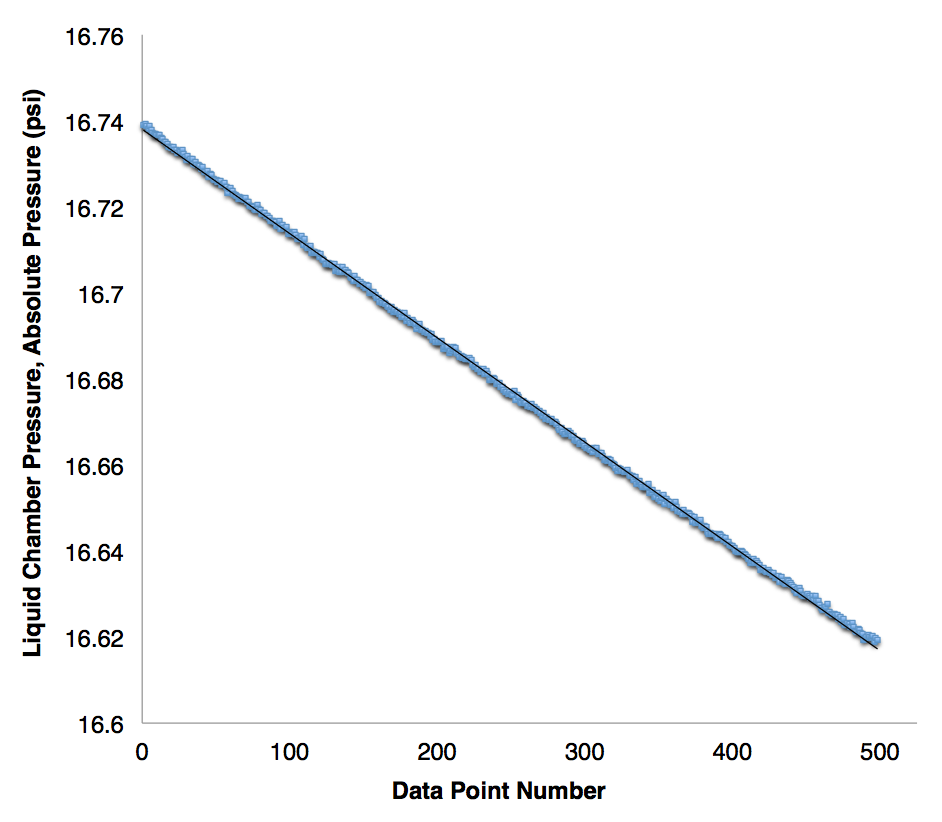

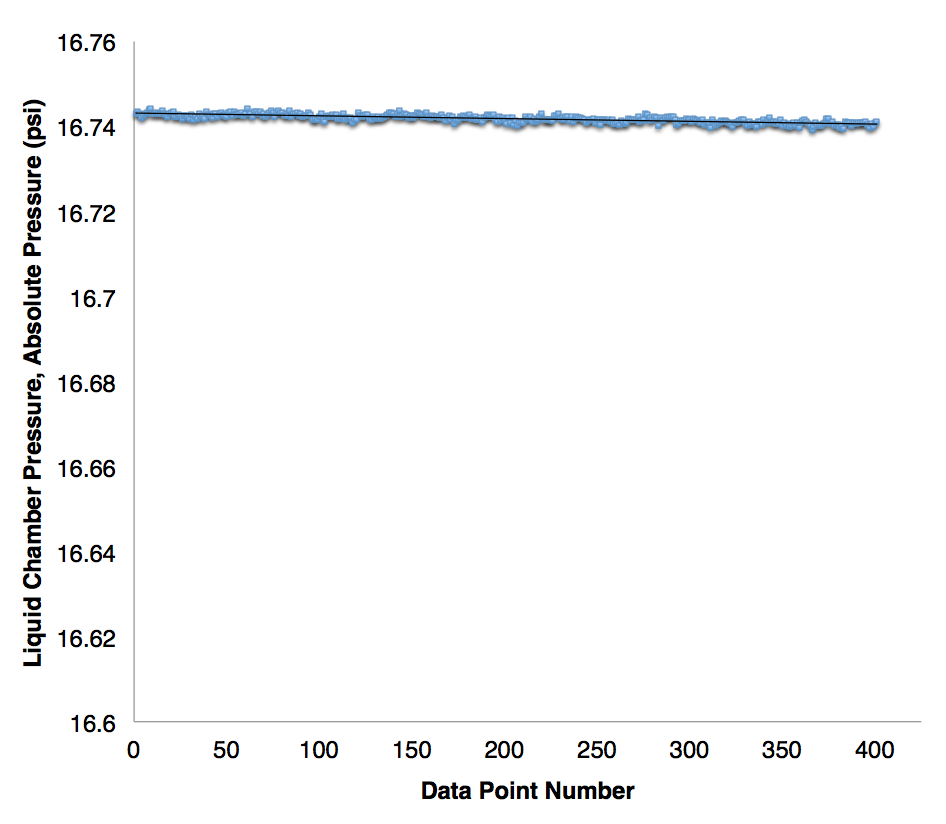


**S1 Fig. Examples of measurement workflow.** Plotted linear fits of parsed data from liquid chamber pressure at full flow (top) and at valve closing at an absolute pressure of 29.66 psi (left) are shown as examples for the measurement workflow. The plotted data is from Sample 1, run 1 of a printed valve with a membrane thickness of 200 μm (it is the first entry in Table S1 below). For the full flow rate as seen in the top plot, the fitted linear trendline has a slope of -2.458E-04 psi per data point, with a R^2^ value of 0.9996. For the bottom plot, the fitted trendline slope is -5.380E-06 psi per data point, with a R^2^ value of 0.5731 (a low R^2^ value as expected due to valve closure resulting in a near-horizontal trendline). Graph pressures plotted as absolute values.

**S1 Table. Data Processing for Flow Rates for Membrane Thickness.**

| Membrane Thickness | Average Applied Control Pressure, Absolute Pressure (psi) | Flow Rate (μL/second) | Change in Flow Pressure, Trendline Slope (psi per data point) | Pressure Change Trendline Fit (R^2^ value) | Applied Control Pressure Standard Deviation (psi) |
| --- | --- | --- | --- | --- | --- |
| 200 μm  Sample 1, Run 1 | 14.51 | 43.31 | -2.458E-04 | 0.9996 | 0.06 |
|  | 19.84 | 30.87 | -1.752E-04 | 0.9990 | 0.18 |
|  | 24.72 | 1.93 | -1.093E-05 | 0.8881 | 0.13 |
|  | 29.66 | 0.95 | -5.380E-06 | 0.5731 | 0.16 |
|  | 14.37 | 38.34 | -2.176E-04 | 0.9991 | 0.06 |
| 200 μm  Sample 1, Run 2 | 14.49 | 42.17 | -2.393E-04 | 0.9993 | 0.06 |
|  | 19.76 | 31.93 | -1.812E-04 | 0.9988 | 0.19 |
|  | 24.76 | 1.65 | -9.367E-06 | 0.8337 | 0.06 |
|  | 29.79 | 1.39 | -7.906E-06 | 0.5911 | 0.05 |
|  | 14.88 | 42.98 | -2.439E-04 | 0.9993 | 0.05 |
| 200 μm  Sample 2, Run 1 | 14.37 | 49.23 | -2.794E-04 | 0.9988 | 0.06 |
|  | 19.56 | 49.53 | -2.811E-04 | 0.9991 | 0.05 |
|  | 24.71 | 43.27 | -2.456E-04 | 0.9989 | 0.05 |
|  | 29.69 | 15.13 | -8.587E-05 | 0.9952 | 0.05 |
|  | 34.54 | 1.38 | -7.836E-06 | 0.8575 | 0.05 |
|  | 14.38 | 47.19 | -2.678E-04 | 0.9993 | 0.06 |
| 200 μm  Sample 2, Run 2 | 14.38 | 49.18 | -2.791E-04 | 0.9980 | 0.06 |
|  | 19.60 | 46.88 | -2.661E-04 | 0.9995 | 0.06 |
|  | 24.47 | 45.77 | -2.598E-04 | 0.9987 | 0.05 |
|  | 29.57 | 22.01 | -1.250E-04 | 0.9961 | 0.04 |
|  | 34.52 | 0.87 | -4.911E-06 | 0.4560 | 0.03 |
| 300 μm  Sample 1, Run 1 | 14.36 | 41.34 | -2.347E-04 | 0.9942 | 0.05 |
|  | 19.71 | 45.50 | -2.582E-04 | 0.9987 | 0.18 |
|  | 24.66 | 48.31 | -2.742E-04 | 0.9955 | 0.05 |
|  | 29.73 | 43.17 | -2.450E-04 | 0.9980 | 0.06 |
|  | 34.60 | 39.69 | -2.253E-04 | 0.9966 | 0.05 |
|  | 39.80 | 3.16 | -1.794E-05 | 0.7771 | 0.04 |
|  | 44.42 | 2.37 | -1.346E-05 | 0.6539 | 0.05 |
|  | 14.37 | 43.22 | -2.453E-04 | 0.9931 | 0.01 |
| 300 μm  Sample 1, Run 2 | 19.67 | 45.91 | -2.606E-04 | 0.9987 | 0.18 |
|  | 24.66 | 44.97 | -2.553E-04 | 0.9986 | 0.06 |
|  | 29.75 | 42.09 | -2.389E-04 | 0.9989 | 0.05 |
|  | 34.59 | 36.18 | -2.054E-04 | 0.9982 | 0.05 |
|  | 39.69 | 11.10 | -6.301E-05 | 0.9939 | 0.04 |
|  | 44.66 | 1.26 | -7.139E-06 | 0.5153 | 0.04 |
|  | 14.37 | 41.67 | -2.365E-04 | 0.9979 | 0.06 |
| 300 μm  Sample 2, Run 1 | 14.33 | 45.20 | -2.566E-04 | 0.9978 | 0.06 |
|  | 19.69 | 51.96 | -2.949E-04 | 0.9963 | 0.06 |
|  | 24.63 | 45.70 | -2.594E-04 | 0.9977 | 0.05 |
|  | 34.48 | 42.52 | -2.413E-04 | 0.9976 | 0.05 |
|  | 39.60 | 29.82 | -1.693E-04 | 0.9975 | 0.03 |
|  | 44.58 | 3.49 | -1.981E-05 | 0.9094 | 0.04 |
|  | 14.32 | 40.31 | -2.288E-04 | 0.9996 | 0.05 |
| 300 μm  Sample 2, Run 2 | 14.32 | 45.84 | -2.602E-04 | 0.9988 | 0.06 |
|  | 19.55 | 47.47 | -2.694E-04 | 0.9969 | 0.05 |
|  | 24.52 | 47.27 | -2.683E-04 | 0.9971 | 0.05 |
|  | 29.62 | 44.03 | -2.499E-04 | 0.9983 | 0.05 |
|  | 34.64 | 40.19 | -2.281E-04 | 0.9978 | 0.03 |
|  | 39.52 | 24.00 | -1.362E-04 | 0.9977 | 0.05 |
|  | 44.51 | 0.94 | -5.309E-06 | 0.6425 | 0.05 |
|  | 14.32 | 41.90 | -2.378E-04 | 0.9993 | 0.05 |
| 400 μm  Sample 1, Run 1 | 14.36 | 44.34 | -2.517E-04 | 0.9989 | 0.06 |
|  | 24.58 | 43.04 | -2.443E-04 | 0.9909 | 0.05 |
|  | 34.67 | 45.06 | -2.557E-04 | 0.9974 | 0.05 |
|  | 44.69 | 37.14 | -2.108E-04 | 0.9991 | 0.04 |
|  | 49.72 | 15.51 | -8.803E-05 | 0.9913 | 0.04 |
|  | 54.62 | 1.59 | -9.040E-06 | 0.6907 | 0.05 |
|  | 14.37 | 40.71 | -2.311E-04 | 0.9978 | 0.06 |
| 400 μm  Sample 1, Run 2 | 14.36 | 43.79 | -2.486E-04 | 0.9996 | 0.06 |
|  | 24.64 | 46.43 | -2.635E-04 | 0.9978 | 0.06 |
|  | 34.61 | 43.65 | -2.477E-04 | 0.9993 | 0.05 |
|  | 44.67 | 36.90 | -2.094E-04 | 0.9983 | 0.03 |
|  | 49.71 | 1.77 | -1.002E-05 | 0.8208 | 0.05 |
|  | 54.56 | 0.79 | -4.469E-06 | 0.4076 | 0.05 |
|  | 14.36 | 40.99 | -2.327E-04 | 0.9989 | 0.05 |
| 400 μm  Sample 2, Run 1 | 14.36 | 47.42 | -2.691E-04 | 0.9990 | 0.06 |
|  | 19.72 | 46.08 | -2.616E-04 | 0.9988 | 0.06 |
|  | 24.61 | 48.74 | -2.766E-04 | 0.9955 | 0.04 |
|  | 29.66 | 46.24 | -2.624E-04 | 0.9977 | 0.04 |
|  | 34.58 | 44.21 | -2.510E-04 | 0.9968 | 0.05 |
|  | 39.52 | 40.61 | -2.305E-04 | 0.9978 | 0.06 |
|  | 44.58 | 30.83 | -1.750E-04 | 0.9963 | 0.04 |
|  | 49.68 | 0.58 | -3.279E-06 | 0.2463 | 0.05 |
|  | 14.36 | 45.15 | -2.563E-04 | 0.9977 | 0.05 |
| 400 μm  Sample 2, Run 2 | 14.35 | 45.59 | -2.587E-04 | 0.9981 | 0.05 |
|  | 19.66 | 48.42 | -2.748E-04 | 0.9980 | 0.05 |
|  | 24.69 | 46.49 | -2.639E-04 | 0.9981 | 0.05 |
|  | 29.58 | 45.08 | -2.559E-04 | 0.9979 | 0.05 |
|  | 34.61 | 44.60 | -2.531E-04 | 0.9977 | 0.05 |
|  | 39.61 | 39.74 | -2.256E-04 | 0.9981 | 0.05 |
|  | 44.58 | 26.51 | -1.505E-04 | 0.9986 | 0.04 |
|  | 49.67 | 0.64 | -3.637E-06 | 0.2716 | 0.05 |
|  | 14.36 | 42.58 | -2.417E-04 | 0.9975 | 0.06 |
| 500 μm  Sample 1, Run 1 | 14.37 | 44.26 | -2.512E-04 | 0.9957 | 0.06 |
|  | 19.78 | 45.42 | -2.578E-04 | 0.9969 | 0.06 |
|  | 24.60 | 42.92 | -2.436E-04 | 0.9988 | 0.06 |
|  | 29.70 | 42.64 | -2.420E-04 | 0.9980 | 0.32 |
|  | 34.64 | 43.03 | -2.442E-04 | 0.9970 | 0.04 |
|  | 39.70 | 40.76 | -2.314E-04 | 0.9983 | 0.04 |
|  | 44.45 | 35.51 | -2.016E-04 | 0.9962 | 0.04 |
|  | 49.74 | 31.39 | -1.782E-04 | 0.9927 | 0.02 |
|  | 54.63 | 0.77 | -4.358E-06 | 0.2640 | 0.05 |
|  | 59.61 | 1.19 | -6.753E-06 | 0.4116 | 0.05 |
|  | 14.37 | 39.71 | -2.254E-04 | 0.9925 | 0.07 |
| 500 μm  Sample 1, Run 2 | 14.36 | 43.92 | -2.493E-04 | 0.9985 | 0.05 |
|  | 24.39 | 41.10 | -2.333E-04 | 0.9990 | 0.05 |
|  | 34.65 | 39.07 | -2.218E-04 | 0.9992 | 0.04 |
|  | 44.69 | 35.38 | -2.008E-04 | 0.9995 | 0.04 |
|  | 49.58 | 13.57 | -7.703E-05 | 0.9979 | 0.05 |
|  | 54.58 | 0.83 | -4.715E-06 | 0.2517 | 0.03 |
|  | 59.58 | 0.54 | -3.063E-06 | 0.1744 | 0.05 |
|  | 14.37 | 39.67 | -2.252E-04 | 0.9978 | 0.05 |
| 500 μm  Sample 2, Run 1 | 14.36 | 48.05 | -2.727E-04 | 0.9983 | 0.05 |
|  | 24.54 | 45.79 | -2.599E-04 | 0.9987 | 0.06 |
|  | 34.51 | 44.76 | -2.540E-04 | 0.9994 | 0.14 |
|  | 44.68 | 42.25 | -2.398E-04 | 0.9990 | 0.04 |
|  | 49.79 | 40.07 | -2.274E-04 | 0.9997 | 0.08 |
|  | 54.50 | 37.25 | -2.114E-04 | 0.9965 | 0.05 |
|  | 59.36 | 0.70 | -3.978E-06 | 0.6148 | 0.05 |
| 500 μm  Sample 2, Run 2 | 14.38 | 46.84 | -2.658E-04 | 0.9990 | 0.06 |
|  | 19.44 | 47.68 | -2.706E-04 | 0.9988 | 0.05 |
|  | 24.61 | 45.13 | -2.562E-04 | 0.9990 | 0.07 |
|  | 29.70 | 45.19 | -2.565E-04 | 0.9952 | 0.05 |
|  | 34.49 | 44.13 | -2.505E-04 | 0.9988 | 0.05 |
|  | 39.70 | 43.54 | -2.471E-04 | 0.9988 | 0.08 |
|  | 44.45 | 41.15 | -2.336E-04 | 0.9992 | 0.02 |
|  | 49.68 | 38.53 | -2.187E-04 | 0.9985 | 0.05 |
|  | 54.38 | 8.82 | -5.007E-05 | 0.9962 | 0.03 |
|  | 59.18 | 0.73 | -4.167E-06 | 0.4694 | 0.09 |
|  | 14.38 | 42.64 | -2.420E-04 | 0.9992 | 0.06 |

**S2 Table. Data Processing for Flow Rates for Channel Width**

| Channel Width | Applied Control Pressure, Absolute Pressure (psi) | Flow Rate (μL/second) | Change in Flow Pressure, Trendline Slope (psi per data point) | Pressure Change Trendline Fit (R^2^ value) | Applied Control Pressure Standard Deviation (psi) |
| --- | --- | --- | --- | --- | --- |
| 700 μm  Sample 1, Run 1 | 14.34 | 44.67 | -2.536E-04 | 0.9990 | 0.05 |
|  | 24.41 | 45.61 | -2.589E-04 | 0.9993 | 0.13 |
|  | 34.48 | 43.19 | -2.451E-04 | 0.9994 | 0.05 |
|  | 44.23 | 26.30 | -1.493E-04 | 0.9949 | 0.03 |
|  | 49.62 | 0.75 | -4.281E-06 | 0.4517 | 0.03 |
|  | 14.34 | 42.37 | -2.405E-04 | 0.9995 | 0.07 |
| 700 μm  Sample 1, Run 2 | 14.34 | 45.92 | -2.607E-04 | 0.9985 | 0.05 |
|  | 19.55 | 48.56 | -2.756E-04 | 0.9976 | 0.06 |
|  | 24.47 | 47.93 | -2.721E-04 | 0.9968 | 0.18 |
|  | 29.58 | 44.92 | -2.550E-04 | 0.9983 | 0.05 |
|  | 34.51 | 42.59 | -2.417E-04 | 0.9990 | 0.04 |
|  | 39.51 | 39.23 | -2.227E-04 | 0.9986 | 0.05 |
|  | 44.41 | 27.12 | -1.540E-04 | 0.9962 | 0.06 |
|  | 49.53 | 0.48 | -2.697E-06 | 0.5002 | 0.04 |
|  | 14.34 | 40.23 | -2.283E-04 | 0.9995 | 0.05 |
| 700 μm  Sample 2, Run 1 | 14.38 | 50.05 | -2.841E-04 | 0.9983 | 0.06 |
|  | 19.47 | 48.43 | -2.749E-04 | 0.9989 | 0.13 |
|  | 24.71 | 47.56 | -2.699E-04 | 0.9992 | 0.04 |
|  | 29.69 | 46.33 | -2.630E-04 | 0.9985 | 0.05 |
|  | 34.56 | 38.88 | -2.207E-04 | 0.9995 | 0.07 |
|  | 39.67 | 0.82 | -4.667E-06 | 0.3652 | 0.05 |
|  | 44.28 | 0.67 | -3.810E-06 | 0.1048 | 0.06 |
|  | 14.38 | 43.60 | -2.475E-04 | 0.9996 | 0.06 |
| 700 μm  Sample 2, Run | 14.38 | 49.16 | -2.790E-04 | 0.9994 | 0.05 |
|  | 19.49 | 47.14 | -2.675E-04 | 0.9996 | 0.22 |
|  | 24.66 | 46.52 | -2.641E-04 | 0.9976 | 0.05 |
|  | 29.68 | 45.49 | -2.582E-04 | 0.9990 | 0.04 |
|  | 34.89 | 35.21 | -1.999E-04 | 0.9995 | 0.06 |
|  | 39.62 | 0.56 | -3.174E-06 | 0.2190 | 0.05 |
|  | 44.72 | 1.07 | -6.102E-06 | 0.2415 | 0.05 |
|  | 14.39 | 44.90 | -2.549E-04 | 0.9995 | 0.06 |
| 900 μm  Sample 1, Run 1 | 14.32 | 46.13 | -2.618E-04 | 0.9982 | 0.05 |
|  | 19.65 | 50.46 | -2.864E-04 | 0.9980 | 0.05 |
|  | 24.48 | 46.20 | -2.622E-04 | 0.9985 | 0.04 |
|  | 34.54 | 33.14 | -1.881E-04 | 0.9988 | 0.04 |
|  | 39.56 | 0.93 | -5.275E-06 | 0.4690 | 0.05 |
|  | 14.33 | 42.95 | -2.438E-04 | 0.9989 | 0.06 |
| 900 μm  Sample 1, Run 2 | 14.37 | 49.01 | -2.782E-04 | 0.9949 | 0.08 |
|  | 19.58 | 46.61 | -2.645E-04 | 0.9985 | 0.06 |
|  | 24.44 | 46.63 | -2.647E-04 | 0.9987 | 0.06 |
|  | 29.59 | 46.06 | -2.614E-04 | 0.9978 | 0.05 |
|  | 34.45 | 22.94 | -1.302E-04 | 0.9979 | 0.05 |
|  | 39.58 | 0.90 | -5.136E-06 | 0.4744 | 0.04 |
|  | 14.32 | 42.85 | -2.432E-04 | 0.9992 | 0.05 |
| 900 μm  Sample 2, Run 1 | 14.36 | 47.25 | -2.682E-04 | 0.9986 | 0.06 |
|  | 19.62 | 46.86 | -2.660E-04 | 0.9991 | 0.05 |
|  | 24.29 | 46.48 | -2.638E-04 | 0.9988 | 0.05 |
|  | 29.58 | 42.49 | -2.412E-04 | 0.9989 | 0.05 |
|  | 34.55 | 36.75 | -2.086E-04 | 0.9988 | 0.05 |
|  | 39.59 | 7.96 | -4.518E-05 | 0.9956 | 0.02 |
|  | 44.59 | 1.17 | -6.642E-06 | 0.4807 | 0.05 |
|  | 14.36 | 42.20 | -2.395E-04 | 0.9995 | 0.06 |
| 900 μm  Sample 2, Run 2 | 14.37 | 45.83 | -2.601E-04 | 0.9997 | 0.05 |
|  | 19.63 | 45.03 | -2.556E-04 | 0.9990 | 0.05 |
|  | 24.52 | 45.28 | -2.570E-04 | 0.9984 | 0.05 |
|  | 29.58 | 40.43 | -2.295E-04 | 0.9989 | 0.04 |
|  | 34.31 | 29.25 | -1.660E-04 | 0.9952 | 0.04 |
|  | 39.62 | 1.09 | -6.194E-06 | 0.5730 | 0.05 |
|  | 44.57 | 0.15 | -8.443E-07 | 0.0079 | 0.03 |
|  | 14.36 | 41.66 | -2.365E-04 | 0.9989 | 0.05 |
| 1100 μm  Sample 1, Run 1 | 14.33 | 48.37 | -2.745E-04 | 0.9969 | 0.06 |
|  | 24.51 | 48.42 | -2.748E-04 | 0.9968 | 0.05 |
|  | 29.68 | 48.11 | -2.731E-04 | 0.9963 | 0.03 |
|  | 34.63 | 28.38 | -1.611E-04 | 0.9986 | 0.03 |
|  | 39.67 | 0.73 | -4.146E-06 | 0.2764 | 0.05 |
|  | 14.32 | 42.14 | -2.392E-04 | 0.9994 | 0.06 |
| 1100 μm  Sample 1, Run 2 | 14.31 | 46.12 | -2.618E-04 | 0.9997 | 0.05 |
|  | 19.67 | 47.00 | -2.668E-04 | 0.9969 | 0.06 |
|  | 24.58 | 47.50 | -2.696E-04 | 0.9961 | 0.05 |
|  | 29.65 | 43.37 | -2.462E-04 | 0.9974 | 0.05 |
|  | 34.57 | 28.27 | -1.605E-04 | 0.9795 | 0.05 |
|  | 39.58 | 0.58 | -3.313E-06 | 0.3794 | 0.03 |
|  | 14.32 | 41.62 | -2.362E-04 | 0.9997 | 0.06 |
| 1100 μm  Sample 2, Run 1 | 14.38 | 49.95 | -2.835E-04 | 0.9987 | 0.06 |
|  | 19.72 | 52.33 | -2.970E-04 | 0.9986 | 0.05 |
|  | 24.71 | 49.47 | -2.808E-04 | 0.9989 | 0.04 |
|  | 29.69 | 46.87 | -2.660E-04 | 0.9988 | 0.04 |
|  | 34.49 | 27.21 | -1.545E-04 | 0.9979 | 0.05 |
|  | 39.72 | 0.53 | -2.995E-06 | 0.2857 | 0.04 |
|  | 14.37 | 42.33 | -2.402E-04 | 0.9997 | 0.06 |
| 1100 μm  Sample 2, Run 2 | 14.38 | 50.77 | -2.882E-04 | 0.9996 | 0.06 |
|  | 19.75 | 49.07 | -2.785E-04 | 0.9994 | 0.05 |
|  | 24.60 | 47.24 | -2.681E-04 | 0.9995 | 0.04 |
|  | 29.56 | 43.69 | -2.480E-04 | 0.9988 | 0.12 |
|  | 34.50 | 22.44 | -1.274E-04 | 0.9967 | 0.05 |
|  | 39.71 | 0.52 | -2.929E-06 | 0.1880 | 0.04 |
|  | 14.38 | 44.92 | -2.549E-04 | 0.9989 | 0.06 |
| 1300 μm  Sample 1, Run 1 | 14.35 | 43.98 | -2.496E-04 | 0.9969 | 0.06 |
|  | 19.67 | 47.91 | -2.720E-04 | 0.9980 | 0.05 |
|  | 24.50 | 45.29 | -2.571E-04 | 0.9980 | 0.04 |
|  | 29.56 | 28.69 | -1.629E-04 | 0.9979 | 0.04 |
|  | 34.50 | 1.08 | -6.111E-06 | 0.4479 | 0.05 |
|  | 39.70 | 3.93 | -2.231E-05 | 0.7247 | 0.04 |
|  | 14.34 | 43.60 | -2.475E-04 | 0.9989 | 0.05 |
| 1300 μm  Sample 1, Run 2 | 14.34 | 47.99 | -2.724E-04 | 0.9979 | 0.05 |
|  | 19.63 | 46.62 | -2.646E-04 | 0.9970 | 0.03 |
|  | 24.37 | 44.80 | -2.543E-04 | 0.9991 | 0.04 |
|  | 29.60 | 29.71 | -1.686E-04 | 0.9964 | 0.05 |
|  | 34.47 | 0.99 | -5.642E-06 | 0.5994 | 0.06 |
|  | 39.59 | 0.43 | -2.436E-06 | 0.2592 | 0.03 |
|  | 14.33 | 42.69 | -2.423E-04 | 0.9975 | 0.05 |
| 1300 μm  Sample 2, Run 1 | 14.36 | 46.88 | -2.661E-04 | 0.9995 | 0.05 |
|  | 19.59 | 49.51 | -2.810E-04 | 0.9981 | 0.06 |
|  | 24.42 | 46.19 | -2.622E-04 | 0.9984 | 0.06 |
|  | 29.51 | 43.29 | -2.457E-04 | 0.9992 | 0.06 |
|  | 34.61 | 14.14 | -8.027E-05 | 0.9922 | 0.04 |
|  | 39.60 | 1.39 | -7.877E-06 | 0.4600 | 0.03 |
|  | 14.36 | 42.38 | -2.406E-04 | 0.9992 | 0.06 |
| 1300 μm  Sample 2, Run 2 | 14.36 | 42.94 | -2.438E-04 | 0.9992 | 0.06 |
|  | 19.58 | 44.66 | -2.535E-04 | 0.9990 | 0.06 |
|  | 24.50 | 42.21 | -2.396E-04 | 0.9982 | 0.04 |
|  | 29.52 | 39.27 | -2.229E-04 | 0.9988 | 0.05 |
|  | 34.48 | 14.32 | -8.131E-05 | 0.9827 | 0.05 |
|  | 39.56 | 0.43 | -2.444E-06 | 0.1881 | 0.05 |
|  | 14.36 | 40.82 | -2.317E-04 | 0.9979 | 0.06 |

**S3 Table. Data Processing for Flow Rates for Membrane Material**

| Membrane Shore Value | Applied Control Pressure, Absolute Pressure (psi)) | Flow Rate (μl/second) | Change in Flow Pressure, Trendline Slope (psi per data point) | Pressure Change Trendline Fit (R^2^ value) | Applied Control Pressure Standard Deviation (psi) |
| --- | --- | --- | --- | --- | --- |
| Shore 26-28 Scale A  Sample 1, Run 1 | 14.36 | 41.34 | -2.347E-04 | 0.9942 | 0.05 |
|  | 19.71 | 45.50 | -2.582E-04 | 0.9987 | 0.18 |
|  | 24.66 | 48.31 | -2.742E-04 | 0.9955 | 0.05 |
|  | 29.73 | 43.17 | -2.450E-04 | 0.9980 | 0.06 |
|  | 34.60 | 39.69 | -2.253E-04 | 0.9966 | 0.05 |
|  | 39.80 | 3.16 | -1.794E-05 | 0.7771 | 0.04 |
|  | 44.42 | 2.37 | -1.346E-05 | 0.6539 | 0.05 |
|  | 14.37 | 43.22 | -2.453E-04 | 0.9931 | 0.01 |
| Shore 26-28 Scale A  Sample 1, Run 2 | 19.67 | 45.91 | -2.606E-04 | 0.9987 | 0.18 |
|  | 24.66 | 44.97 | -2.553E-04 | 0.9986 | 0.06 |
|  | 29.75 | 42.09 | -2.389E-04 | 0.9989 | 0.05 |
|  | 34.59 | 36.18 | -2.054E-04 | 0.9982 | 0.05 |
|  | 39.69 | 11.10 | -6.300E-05 | 0.9939 | 0.04 |
|  | 44.66 | 1.26 | -7.140E-06 | 0.5153 | 0.04 |
|  | 14.37 | 41.67 | -2.365E-04 | 0.9979 | 0.06 |
| Shore 26-28 Scale A  Sample 2, Run 1 | 14.33 | 45.20 | -2.566E-04 | 0.9978 | 0.06 |
|  | 19.69 | 44.32 | -2.516E-04 | 0.9989 | 0.06 |
|  | 24.63 | 42.71 | -2.424E-04 | 0.9977 | 0.05 |
|  | 34.48 | 42.52 | -2.413E-04 | 0.9976 | 0.05 |
|  | 39.60 | 29.82 | -1.693E-04 | 0.9975 | 0.03 |
|  | 44.58 | 3.49 | -1.981E-05 | 0.9094 | 0.04 |
|  | 14.32 | 40.31 | -2.288E-04 | 0.9996 | 0.05 |
| Shore 26-28 Scale A  Sample 2, Run 2 | 14.32 | 45.84 | -2.602E-04 | 0.9988 | 0.06 |
|  | 19.55 | 47.47 | -2.694E-04 | 0.9969 | 0.05 |
|  | 24.52 | 47.27 | -2.683E-04 | 0.9971 | 0.05 |
|  | 29.62 | 44.03 | -2.499E-04 | 0.9983 | 0.05 |
|  | 34.64 | 40.19 | -2.281E-04 | 0.9978 | 0.03 |
|  | 39.52 | 24.00 | -1.362E-04 | 0.9977 | 0.05 |
|  | 44.51 | 0.94 | -5.309E-06 | 0.6425 | 0.05 |
|  | 14.32 | 41.90 | -2.378E-04 | 0.9993 | 0.05 |
| Shore 35-40 Scale A  Sample 1, Run 1 | 14.39 | 46.57 | -2.643E-04 | 0.9720 | 0.06 |
|  | 19.69 | 44.23 | -2.510E-04 | 0.9931 | 0.06 |
|  | 24.64 | 40.87 | -2.320E-04 | 0.9985 | 0.05 |
|  | 29.67 | 41.17 | -2.337E-04 | 0.9988 | 0.04 |
|  | 34.61 | 38.75 | -2.199E-04 | 0.9990 | 0.05 |
|  | 39.66 | 34.28 | -1.946E-04 | 0.9977 | 0.05 |
|  | 44.59 | 22.98 | -1.305E-04 | 0.9981 | 0.05 |
|  | 49.59 | 1.23 | -7.002E-06 | 0.5908 | 0.05 |
|  | 14.38 | 37.94 | -2.154E-04 | 0.9995 | 0.06 |
| Shore 35-40 Scale A  Sample 1, Run 2 | 14.38 | 50.23 | -2.851E-04 | 0.9997 | 0.06 |
|  | 19.62 | 47.93 | -2.721E-04 | 0.9990 | 0.05 |
|  | 24.63 | 47.84 | -2.715E-04 | 0.9982 | 0.09 |
|  | 29.60 | 46.89 | -2.662E-04 | 0.9963 | 0.06 |
|  | 34.57 | 44.20 | -2.509E-04 | 0.9972 | 0.05 |
|  | 39.71 | 35.05 | -1.989E-04 | 0.9985 | 0.04 |
|  | 44.36 | 15.65 | -8.887E-05 | 0.9919 | 0.05 |
|  | 49.61 | 1.45 | -8.251E-06 | 0.4896 | 0.04 |
| Shore 57-63 Scale A  Sample 1,  Run 1 | 14.37 | 41.25 | -2.342E-04 | 0.9720 | 0.06 |
|  | 44.78 | 40.37 | -2.292E-04 | 0.9931 | 0.06 |
|  | 67.47 | 38.39 | -2.179E-04 | 0.9985 | 0.05 |
|  | 87.75 | 20.10 | -1.141E-04 | 0.9988 | 0.04 |
|  | 14.38 | 51.32 | -2.913E-04 | 0.9990 | 0.05 |
