# Supplementary figures and images for: 3D Printed Multimaterial Microfluidic Valve
Source: PLoS One. 2016 Aug 15;11(8):e0160624. doi: 10.1371/journal.pone.0160624 (PMC4985141; doi:10.1371/journal.pone.0160624)

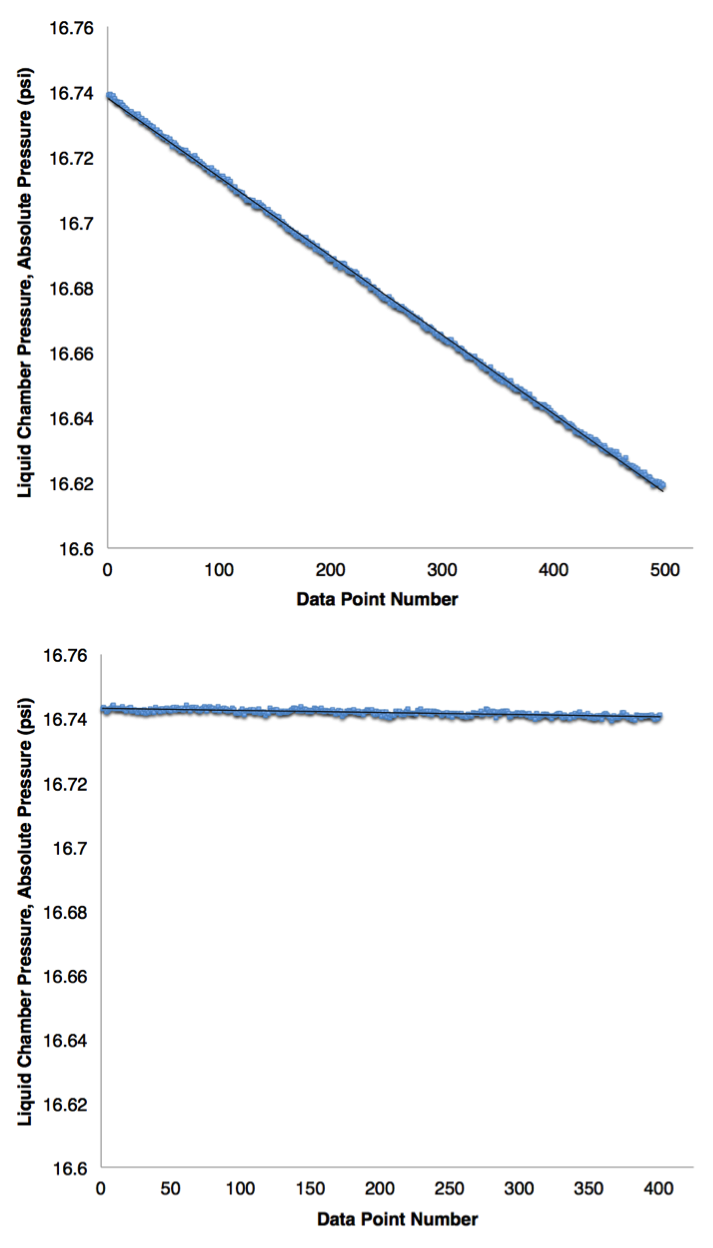

Supplement: S1 Fig — Plotted linear fits of parsed data from liquid chamber pressure at full flow (top) and at valve closing at an absolute pressure of 29.66 psi (left) are shown as examples for the measurement workflow. The plotted data is from Sample 1, run 1 of a printed valve with a membrane thickness of 200 μm (it is the first entry in S1 Table below). For the full flow rate as seen in the top plot, the fitted linear trendline has a slope of -2.458E-04 psi per data point, with a R2 value of 0.9996. For the bottom plot, the fitted trendline slope is -5.380E-06 psi per data point, with a R2 value of 0.5731 (a low R2 value as expected due to valve closure resulting in a near-horizontal trendline). Graph pressures plotted as absolute values. (TIF) [file pone.0160624.s003.tif]
