# Supplementary material for: 3D Printed Multimaterial Microfluidic Valve
Source: PLoS One. 2016 Aug 15;11(8):e0160624. doi: 10.1371/journal.pone.0160624 (PMC4985141; doi:10.1371/journal.pone.0160624)
Supplement: S1 Table — (PDF) [file pone.0160624.s004.pdf]

S1 Table. Data Processing for Flow Rates for Membrane Thickness.

Membrane Thickness	Average Applied Control Pressure, Absolute Pressure (psi)	Flow Rate (µL/second)	Change in Flow Pressure, Trendline Slope (psi per data point)	Pressure Change Trendline Fit (R^2 value)	Applied Control Pressure Standard Deviation (psi)
200 µm Sample 1, Run 1	14.51	43.31	-2.458E-04	0.9996	0.06
	19.84	30.87	-1.752E-04	0.9990	0.18
	24.72	1.93	-1.093E-05	0.8881	0.13
	29.66	0.95	-5.380E-06	0.5731	0.16
	14.37	38.34	-2.176E-04	0.9991	0.06
200 µm Sample 1, Run 2	14.49	42.17	-2.393E-04	0.9993	0.06
	19.76	31.93	-1.812E-04	0.9988	0.19
	24.76	1.65	-9.367E-06	0.8337	0.06
	29.79	1.39	-7.906E-06	0.5911	0.05
	14.88	42.98	-2.439E-04	0.9993	0.05
200 µm Sample 2, Run 1	14.37	49.23	-2.794E-04	0.9988	0.06
	19.56	49.53	-2.811E-04	0.9991	0.05
	24.71	43.27	-2.456E-04	0.9989	0.05
	29.69	15.13	-8.587E-05	0.9952	0.05
	34.54	1.38	-7.836E-06	0.8575	0.05
	14.38	47.19	-2.678E-04	0.9993	0.06
200 µm Sample 2, Run 2	14.38	49.18	-2.791E-04	0.9980	0.06
	19.60	46.88	-2.661E-04	0.9995	0.06
	24.47	45.77	-2.598E-04	0.9987	0.05
	29.57	22.01	-1.250E-04	0.9961	0.04
	34.52	0.87	-4.911E-06	0.4560	0.03
300 µm Sample 1, Run 1	14.36	41.34	-2.347E-04	0.9942	0.05
	19.71	45.50	-2.582E-04	0.9987	0.18
	24.66	48.31	-2.742E-04	0.9955	0.05
	29.73	43.17	-2.450E-04	0.9980	0.06
	34.60	39.69	-2.253E-04	0.9966	0.05
	39.80	3.16	-1.794E-05	0.7771	0.04
	44.42	2.37	-1.346E-05	0.6539	0.05
	14.37	43.22	-2.453E-04	0.9931	0.01
300 µm Sample 1, Run 2	19.67	45.91	-2.606E-04	0.9987	0.18
	24.66	44.97	-2.553E-04	0.9986	0.06
	29.75	42.09	-2.389E-04	0.9989	0.05
	34.59	36.18	-2.054E-04	0.9982	0.05
	39.69	11.10	-6.301E-05	0.9939	0.04
	44.66	1.26	-7.139E-06	0.5153	0.04

300 µm Sample 2, Run 1	14.37	41.67	-2.365E-04	0.9979	0.06
	14.33	45.20	-2.566E-04	0.9978	0.06
	19.69	51.96	-2.949E-04	0.9963	0.06
	24.63	45.70	-2.594E-04	0.9977	0.05
	34.48	42.52	-2.413E-04	0.9976	0.05
	39.60	29.82	-1.693E-04	0.9975	0.03
	44.58	3.49	-1.981E-05	0.9094	0.04
300 µm Sample 2, Run 2	14.32	40.31	-2.288E-04	0.9996	0.05
	14.32	45.84	-2.602E-04	0.9988	0.06
	19.55	47.47	-2.694E-04	0.9969	0.05
	24.52	47.27	-2.683E-04	0.9971	0.05
	29.62	44.03	-2.499E-04	0.9983	0.05
	34.64	40.19	-2.281E-04	0.9978	0.03
	39.52	24.00	-1.362E-04	0.9977	0.05
	44.51	0.94	-5.309E-06	0.6425	0.05
400 µm Sample 1, Run 1	14.32	41.90	-2.378E-04	0.9993	0.05
	14.36	44.34	-2.517E-04	0.9989	0.06
	24.58	43.04	-2.443E-04	0.9909	0.05
	34.67	45.06	-2.557E-04	0.9974	0.05
	44.69	37.14	-2.108E-04	0.9991	0.04
	49.72	15.51	-8.803E-05	0.9913	0.04
	54.62	1.59	-9.040E-06	0.6907	0.05
400 µm Sample 1, Run 2	14.37	40.71	-2.311E-04	0.9978	0.06
	14.36	43.79	-2.486E-04	0.9996	0.06
	24.64	46.43	-2.635E-04	0.9978	0.06
	34.61	43.65	-2.477E-04	0.9993	0.05
	44.67	36.90	-2.094E-04	0.9983	0.03
	49.71	1.77	-1.002E-05	0.8208	0.05
	54.56	0.79	-4.469E-06	0.4076	0.05
400 µm Sample 2, Run 1	14.36	40.99	-2.327E-04	0.9989	0.05
	14.36	47.42	-2.691E-04	0.9990	0.06
	19.72	46.08	-2.616E-04	0.9988	0.06
	24.61	48.74	-2.766E-04	0.9955	0.04
	29.66	46.24	-2.624E-04	0.9977	0.04
	34.58	44.21	-2.510E-04	0.9968	0.05
	39.52	40.61	-2.305E-04	0.9978	0.06
	44.58	30.83	-1.750E-04	0.9963	0.04
	49.68	0.58	-3.279E-06	0.2463	0.05
400 µm Sample 2, Run 2	14.36	45.15	-2.563E-04	0.9977	0.05
	14.35	45.59	-2.587E-04	0.9981	0.05
	19.66	48.42	-2.748E-04	0.9980	0.05
	24.69	46.49	-2.639E-04	0.9981	0.05
	29.58	45.08	-2.559E-04	0.9979	0.05

	34.61	44.60	-2.531E-04	0.9977	0.05
	39.61	39.74	-2.256E-04	0.9981	0.05
	44.58	26.51	-1.505E-04	0.9986	0.04
	49.67	0.64	-3.637E-06	0.2716	0.05
	14.36	42.58	-2.417E-04	0.9975	0.06
500 µm Sample 1, Run 1	14.37	44.26	-2.512E-04	0.9957	0.06
	19.78	45.42	-2.578E-04	0.9969	0.06
	24.60	42.92	-2.436E-04	0.9988	0.06
	29.70	42.64	-2.420E-04	0.9980	0.32
	34.64	43.03	-2.442E-04	0.9970	0.04
	39.70	40.76	-2.314E-04	0.9983	0.04
	44.45	35.51	-2.016E-04	0.9962	0.04
	49.74	31.39	-1.782E-04	0.9927	0.02
	54.63	0.77	-4.358E-06	0.2640	0.05
	59.61	1.19	-6.753E-06	0.4116	0.05
	14.37	39.71	-2.254E-04	0.9925	0.07
500 µm Sample 1, Run 2	14.36	43.92	-2.493E-04	0.9985	0.05
	24.39	41.10	-2.333E-04	0.9990	0.05
	34.65	39.07	-2.218E-04	0.9992	0.04
	44.69	35.38	-2.008E-04	0.9995	0.04
	49.58	13.57	-7.703E-05	0.9979	0.05
	54.58	0.83	-4.715E-06	0.2517	0.03
	59.58	0.54	-3.063E-06	0.1744	0.05
	14.37	39.67	-2.252E-04	0.9978	0.05
500 µm Sample 2, Run 1	14.36	48.05	-2.727E-04	0.9983	0.05
	24.54	45.79	-2.599E-04	0.9987	0.06
	34.51	44.76	-2.540E-04	0.9994	0.14
	44.68	42.25	-2.398E-04	0.9990	0.04
	49.79	40.07	-2.274E-04	0.9997	0.08
	54.50	37.25	-2.114E-04	0.9965	0.05
	59.36	0.70	-3.978E-06	0.6148	0.05
500 µm Sample 2, Run 2	14.38	46.84	-2.658E-04	0.9990	0.06
	19.44	47.68	-2.706E-04	0.9988	0.05
	24.61	45.13	-2.562E-04	0.9990	0.07
	29.70	45.19	-2.565E-04	0.9952	0.05
	34.49	44.13	-2.505E-04	0.9988	0.05
	39.70	43.54	-2.471E-04	0.9988	0.08
	44.45	41.15	-2.336E-04	0.9992	0.02
	49.68	38.53	-2.187E-04	0.9985	0.05
	54.38	8.82	-5.007E-05	0.9962	0.03
	59.18	0.73	-4.167E-06	0.4694	0.09
	14.38	42.64	-2.420E-04	0.9992	0.06
