# Supplementary material for: 3D Printed Multimaterial Microfluidic Valve
Source: PLoS One. 2016 Aug 15;11(8):e0160624. doi: 10.1371/journal.pone.0160624 (PMC4985141; doi:10.1371/journal.pone.0160624)
Supplement: S2 Table — (PDF) [file pone.0160624.s005.pdf]

S2 Table. Data Processing for Flow Rates for Channel Width

Channel Width	Applied Control Pressure, Absolute Pressure (psi)	Flow Rate (µL/second)	Change in Flow Pressure, Trendline Slope (psi per data point)	Pressure Change Trendline Fit (R^2 value)	Applied Control Pressure Standard Deviation (psi)
700 µm Sample 1, Run 1	14.34	44.67	-2.536E-04	0.9990	0.05
	24.41	45.61	-2.589E-04	0.9993	0.13
	34.48	43.19	-2.451E-04	0.9994	0.05
	44.23	26.30	-1.493E-04	0.9949	0.03
	49.62	0.75	-4.281E-06	0.4517	0.03
	14.34	42.37	-2.405E-04	0.9995	0.07
700 µm Sample 1, Run 2	14.34	45.92	-2.607E-04	0.9985	0.05
	19.55	48.56	-2.756E-04	0.9976	0.06
	24.47	47.93	-2.721E-04	0.9968	0.18
	29.58	44.92	-2.550E-04	0.9983	0.05
	34.51	42.59	-2.417E-04	0.9990	0.04
	39.51	39.23	-2.227E-04	0.9986	0.05
	44.41	27.12	-1.540E-04	0.9962	0.06
	49.53	0.48	-2.697E-06	0.5002	0.04
	14.34	40.23	-2.283E-04	0.9995	0.05
700 µm Sample 2, Run 1	14.38	50.05	-2.841E-04	0.9983	0.06
	19.47	48.43	-2.749E-04	0.9989	0.13
	24.71	47.56	-2.699E-04	0.9992	0.04
	29.69	46.33	-2.630E-04	0.9985	0.05
	34.56	38.88	-2.207E-04	0.9995	0.07
	39.67	0.82	-4.667E-06	0.3652	0.05
	44.28	0.67	-3.810E-06	0.1048	0.06
	14.38	43.60	-2.475E-04	0.9996	0.06
700 µm Sample 2, Run	14.38	49.16	-2.790E-04	0.9994	0.05
	19.49	47.14	-2.675E-04	0.9996	0.22
	24.66	46.52	-2.641E-04	0.9976	0.05
	29.68	45.49	-2.582E-04	0.9990	0.04
	34.89	35.21	-1.999E-04	0.9995	0.06
	39.62	0.56	-3.174E-06	0.2190	0.05
	44.72	1.07	-6.102E-06	0.2415	0.05
	14.39	44.90	-2.549E-04	0.9995	0.06
900 µm Sample 1, Run 1	14.32	46.13	-2.618E-04	0.9982	0.05
	19.65	50.46	-2.864E-04	0.9980	0.05
	24.48	46.20	-2.622E-04	0.9985	0.04
	34.54	33.14	-1.881E-04	0.9988	0.04
	39.56	0.93	-5.275E-06	0.4690	0.05

900 µm Sample 1, Run 2	14.33	42.95	-2.438E-04	0.9989	0.06
	14.37	49.01	-2.782E-04	0.9949	0.08
	19.58	46.61	-2.645E-04	0.9985	0.06
	24.44	46.63	-2.647E-04	0.9987	0.06
	29.59	46.06	-2.614E-04	0.9978	0.05
	34.45	22.94	-1.302E-04	0.9979	0.05
	39.58	0.90	-5.136E-06	0.4744	0.04
900 µm Sample 2, Run 1	14.32	42.85	-2.432E-04	0.9992	0.05
	14.36	47.25	-2.682E-04	0.9986	0.06
	19.62	46.86	-2.660E-04	0.9991	0.05
	24.29	46.48	-2.638E-04	0.9988	0.05
	29.58	42.49	-2.412E-04	0.9989	0.05
	34.55	36.75	-2.086E-04	0.9988	0.05
	39.59	7.96	-4.518E-05	0.9956	0.02
	44.59	1.17	-6.642E-06	0.4807	0.05
900 µm Sample 2, Run 2	14.36	42.20	-2.395E-04	0.9995	0.06
	14.37	45.83	-2.601E-04	0.9997	0.05
	19.63	45.03	-2.556E-04	0.9990	0.05
	24.52	45.28	-2.570E-04	0.9984	0.05
	29.58	40.43	-2.295E-04	0.9989	0.04
	34.31	29.25	-1.660E-04	0.9952	0.04
	39.62	1.09	-6.194E-06	0.5730	0.05
	44.57	0.15	-8.443E-07	0.0079	0.03
1100 µm Sample 1, Run 1	14.36	41.66	-2.365E-04	0.9989	0.05
	14.33	48.37	-2.745E-04	0.9969	0.06
	24.51	48.42	-2.748E-04	0.9968	0.05
	29.68	48.11	-2.731E-04	0.9963	0.03
	34.63	28.38	-1.611E-04	0.9986	0.03
	39.67	0.73	-4.146E-06	0.2764	0.05
1100 µm Sample 1, Run 2	14.32	42.14	-2.392E-04	0.9994	0.06
	14.31	46.12	-2.618E-04	0.9997	0.05
	19.67	47.00	-2.668E-04	0.9969	0.06
	24.58	47.50	-2.696E-04	0.9961	0.05
	29.65	43.37	-2.462E-04	0.9974	0.05
	34.57	28.27	-1.605E-04	0.9795	0.05
	39.58	0.58	-3.313E-06	0.3794	0.03
1100 µm Sample 2, Run 1	14.32	41.62	-2.362E-04	0.9997	0.06
	14.38	49.95	-2.835E-04	0.9987	0.06
	19.72	52.33	-2.970E-04	0.9986	0.05
	24.71	49.47	-2.808E-04	0.9989	0.04
	29.69	46.87	-2.660E-04	0.9988	0.04
	34.49	27.21	-1.545E-04	0.9979	0.05
	39.72	0.53	-2.995E-06	0.2857	0.04

	14.37	42.33	-2.402E-04	0.9997	0.06
1100 µm Sample 2, Run 2	14.38	50.77	-2.882E-04	0.9996	0.06
	19.75	49.07	-2.785E-04	0.9994	0.05
	24.60	47.24	-2.681E-04	0.9995	0.04
	29.56	43.69	-2.480E-04	0.9988	0.12
	34.50	22.44	-1.274E-04	0.9967	0.05
	39.71	0.52	-2.929E-06	0.1880	0.04
	14.38	44.92	-2.549E-04	0.9989	0.06
1300 µm Sample 1, Run 1	14.35	43.98	-2.496E-04	0.9969	0.06
	19.67	47.91	-2.720E-04	0.9980	0.05
	24.50	45.29	-2.571E-04	0.9980	0.04
	29.56	28.69	-1.629E-04	0.9979	0.04
	34.50	1.08	-6.111E-06	0.4479	0.05
	39.70	3.93	-2.231E-05	0.7247	0.04
	14.34	43.60	-2.475E-04	0.9989	0.05
1300 µm Sample 1, Run 2	14.34	47.99	-2.724E-04	0.9979	0.05
	19.63	46.62	-2.646E-04	0.9970	0.03
	24.37	44.80	-2.543E-04	0.9991	0.04
	29.60	29.71	-1.686E-04	0.9964	0.05
	34.47	0.99	-5.642E-06	0.5994	0.06
	39.59	0.43	-2.436E-06	0.2592	0.03
	14.33	42.69	-2.423E-04	0.9975	0.05
1300 µm Sample 2, Run 1	14.36	46.88	-2.661E-04	0.9995	0.05
	19.59	49.51	-2.810E-04	0.9981	0.06
	24.42	46.19	-2.622E-04	0.9984	0.06
	29.51	43.29	-2.457E-04	0.9992	0.06
	34.61	14.14	-8.027E-05	0.9922	0.04
	39.60	1.39	-7.877E-06	0.4600	0.03
	14.36	42.38	-2.406E-04	0.9992	0.06
1300 µm Sample 2, Run 2	14.36	42.94	-2.438E-04	0.9992	0.06
	19.58	44.66	-2.535E-04	0.9990	0.06
	24.50	42.21	-2.396E-04	0.9982	0.04
	29.52	39.27	-2.229E-04	0.9988	0.05
	34.48	14.32	-8.131E-05	0.9827	0.05
	39.56	0.43	-2.444E-06	0.1881	0.05
	14.36	40.82	-2.317E-04	0.9979	0.06
