# Supplementary material for: 3D Printed Multimaterial Microfluidic Valve
Source: PLoS One. 2016 Aug 15;11(8):e0160624. doi: 10.1371/journal.pone.0160624 (PMC4985141; doi:10.1371/journal.pone.0160624)
Supplement: S3 Table — (PDF) [file pone.0160624.s006.pdf]

S3 Table. Data Processing for Flow Rates for Membrane Material

Membrane Shore Value	Applied Control Pressure, Absolute Pressure (psi))	Flow Rate (μl/second)	Change in Flow Pressure, Trendline Slope (psi per data point)	Pressure Change Trendline Fit (R^2 value)	Applied Control Pressure Standard Deviation (psi)
Shore 26-28 Scale A Sample 1, Run 1	14.36	41.34	-2.347E-04	0.9942	0.05
	19.71	45.50	-2.582E-04	0.9987	0.18
	24.66	48.31	-2.742E-04	0.9955	0.05
	29.73	43.17	-2.450E-04	0.9980	0.06
	34.60	39.69	-2.253E-04	0.9966	0.05
	39.80	3.16	-1.794E-05	0.7771	0.04
	44.42	2.37	-1.346E-05	0.6539	0.05
	14.37	43.22	-2.453E-04	0.9931	0.01
Shore 26-28 Scale A Sample 1, Run 2	19.67	45.91	-2.606E-04	0.9987	0.18
	24.66	44.97	-2.553E-04	0.9986	0.06
	29.75	42.09	-2.389E-04	0.9989	0.05
	34.59	36.18	-2.054E-04	0.9982	0.05
	39.69	11.10	-6.300E-05	0.9939	0.04
	44.66	1.26	-7.140E-06	0.5153	0.04
	14.37	41.67	-2.365E-04	0.9979	0.06
Shore 26-28 Scale A Sample 2, Run 1	14.33	45.20	-2.566E-04	0.9978	0.06
	19.69	44.32	-2.516E-04	0.9989	0.06
	24.63	42.71	-2.424E-04	0.9977	0.05
	34.48	42.52	-2.413E-04	0.9976	0.05
	39.60	29.82	-1.693E-04	0.9975	0.03
	44.58	3.49	-1.981E-05	0.9094	0.04
	14.32	40.31	-2.288E-04	0.9996	0.05
Shore 26-28 Scale A Sample 2, Run 2	14.32	45.84	-2.602E-04	0.9988	0.06
	19.55	47.47	-2.694E-04	0.9969	0.05
	24.52	47.27	-2.683E-04	0.9971	0.05
	29.62	44.03	-2.499E-04	0.9983	0.05
	34.64	40.19	-2.281E-04	0.9978	0.03
	39.52	24.00	-1.362E-04	0.9977	0.05
	44.51	0.94	-5.309E-06	0.6425	0.05
	14.32	41.90	-2.378E-04	0.9993	0.05
Shore 35-40 Scale A Sample 1, Run 1	14.39	46.57	-2.643E-04	0.9720	0.06
	19.69	44.23	-2.510E-04	0.9931	0.06
	24.64	40.87	-2.320E-04	0.9985	0.05
	29.67	41.17	-2.337E-04	0.9988	0.04
	34.61	38.75	-2.199E-04	0.9990	0.05
	39.66	34.28	-1.946E-04	0.9977	0.05

	44.59	22.98	-1.305E-04	0.9981	0.05
	49.59	1.23	-7.002E-06	0.5908	0.05
	14.38	37.94	-2.154E-04	0.9995	0.06
Shore 35-40 Scale A Sample 1, Run 2	14.38	50.23	-2.851E-04	0.9997	0.06
	19.62	47.93	-2.721E-04	0.9990	0.05
	24.63	47.84	-2.715E-04	0.9982	0.09
	29.60	46.89	-2.662E-04	0.9963	0.06
	34.57	44.20	-2.509E-04	0.9972	0.05
	39.71	35.05	-1.989E-04	0.9985	0.04
	44.36	15.65	-8.887E-05	0.9919	0.05
	49.61	1.45	-8.251E-06	0.4896	0.04
Shore 57-63 Scale A Sample 1, Run 1	14.37	41.25	-2.342E-04	0.9720	0.06
	44.78	40.37	-2.292E-04	0.9931	0.06
	67.47	38.39	-2.179E-04	0.9985	0.05
	87.75	20.10	-1.141E-04	0.9988	0.04
	14.38	51.32	-2.913E-04	0.9990	0.05
